# Botanical Products in the Treatment and Control of Schistosomiasis: Recent Studies and Distribution of Active Plant Resources According to Affected Regions

**DOI:** 10.3390/biology9080223

**Published:** 2020-08-13

**Authors:** Ricardo Diego Duarte Galhardo de Albuquerque, Mohamad Fawzi Mahomoodally, Devina Lobine, Shanno Suroowan, Kannan RR Rengasamy

**Affiliations:** 1 Laboratory of Technology in Natural Products, Universidade Federal Fluminense (UFF), Niterói 24241-002, Brazil; ricardo-diego-cf@hotmail.com; 2 Institute of Research and Development, Duy Tan University, Da Nang 550000, Vietnam; f.mahomoodally@uom.ac.mu; 3 Department of Health Sciences, Faculty of Science, University of Mauritius, Réduit 80835, Mauritius; devina.lobine@gmail.com (D.L.); s2thegame@gmail.com (S.S.); 4 Bionanotechnology Research Group, Ton Duc Thang University, Ho Chi Minh City, Vietnam; 5 Faculty of Pharmacy, Ton Duc Thang University, Ho Chi Minh City, Vietnam

**Keywords:** antiparasitics, molluscicide, schistosomiasis, medicinal plants, Africa, Asia, Brazil

## Abstract

Schistosomiasis, a parasitic disease caused by trematodes of the genus *Schistosoma*, is the second most prevalent parasitic disease in the world. It affects around 200 million people. Clinical treatment, prophylaxis, and prevention are performed in countries susceptible to schistosomiasis. In the pharmacological treatment for an acute form of schistosomiasis, the use of antiparasitics, mainly praziquantel, is more common. As an alternative way, prevention methods such as reducing the population of intermediate hosts (mollusks) with molluscicides are important in the control of this disease by interrupting the biological cycle of this etiological parasite. Despite the importance of pharmacological agents and molluscicides, they have side effects and environmental toxicity. In addition, they can lead to the development of resistance enhancing of parasites, and lead to the search for new and effective drugs, including resources of vegetal origin, which in turn, are abundant in the affected countries. Thus, the purpose of this review is to summarize recent studies on botanical products with potential for the control of schistosomiasis, including anti-Schistosoma and molluscicide activities. In addition, species and plant derivatives according to their origin or geographical importance indicating a possible utility of local resources for countries most affected by the disease are presented.

## 1. Introduction

Schistosomiasis is also commonly known as bilharziasis or snail fever. It is a parasitic disease caused by trematodes of the genus *Schistosoma* [1], which in turn are transmitted to humans through their intermediate hosts such as planorbids belonging to genus *Biomphalaria (S. mansoni)*, *Oncomelania* (*S. japonicum*), and *Bulinus* (*S. haematobium*) [2,3]. According to the World Health Organization (WHO), schistosomiasis is the second most prevalent parasitic disease in the world after malaria. It is associated with socioeconomic problems and water supply. It directly affects around 290 million people, with more than 700 million of them in risk areas. It is distributed in 78 countries, with major prevalence in South America, Sub-Saharan Africa, and Asia [4,5]. The worldwide burden associated with schistosomiasis has been projected to be at 2.6 million disability adjusted life years [6].

Main clinical manifestations of this disease include hepatomegaly, splenomegaly, periportal fibrosis, and appendicitis. An investigation for the different regions of Africa has revealed a high prevalence of infection [7]. In Mbita and the islands close to Lake Victoria, the prevalence of this disease in school children aged 5 to 19 years was 60.5%. In Lake Rweru, Rwanda, 21.1% of inhabitants were infected [8]. One study has been conducted in Nigeria to document the proportion of pregnant women affected by this disease. It was found that an astonishing 20.8% of women aged 15 to 42 years were infected by this parasite [9]. In areas where the disease is endemic, it is one of the main causes of pulmonary hypertension [10]. The prevalence of this disease in different African regions is summarized in Table 1.

The transmission of schistosomiasis mainly depends on the presence of the infected person and faecal or urinary release of eggs from helminths into water environments containing the host mollusk, thus maintaining the life cycle of the parasite [4]. Globally, there are six species of schistosomes that can infect a human, including *Schistosoma mansoni*, *Schistosoma japonicum*, *Schistosoma haematobium* and, to a lesser extension, Schistosoma intercalatum, Schistosoma mekongi, and *Schistosoma guineesis* [17].

In the case of *S. mansoni* and *S. japonicum* infections, the acute form of this disease causes symptoms such as fever, myalgia, fatigue, malaise, dry cough, bloody mucus, diarrhea, diffuse abdominal pain, hepatosplenomegaly, eosinophilia, and the release of viable *Schistosoma* eggs in faeces, whereas dysuria, painful hematuria, urinary obstruction, vaginal discharge, or pain/bleeding after intercourse and the release of viable eggs in urine occurs in infections caused by *S. haematobium*. The chronic stage is caused by an egg deposition by individuals of the genus *Schistosoma* and reactions of the host’s immune system. The fundamental characteristic of this form in *S. mansoni* and *S. japonicum* infections is the development of portal hypertension, leading to splenomegaly, hepatosplenic, and hepatointestinal forms. Varicose veins of the esophagus, spleen enlargement, hepatic cirrhosis, and urinary, intestinal, hepatic, and ectopic forms of the disease are other symptoms that may appear at this stage of the disease. In the infections caused by *S. haematobium*, the chronic stage is initiated by lodged eggs in the urogenital system, causing granulomatous host response and subsequent tissue inflammation [18].

Another form of the disease is neuroeschistosomiasis, a more frequent and disabling ectopic form compromising the central nervous system. Its diagnosis is based on epidemiological, clinical, and laboratory data. In some countries such as Brazil, this form of the disease has shown a considerable increase in the last two decades [19].

Clinical treatment, prophylaxis, and prevention are usually performed in countries susceptible to schistosomiasis. In the pharmacological treatment of acute schistosomiasis, the use of oxamniquine and praziquantel, often associated, is common. Prevention methods such as reducing the population of intermediate hosts (mollusks) are also important in the control of the disease since they can interrupt the biological cycle of the etiological parasite [20,21].

## 2. Genus *Schistosoma* and Its Biological Cycle Importance in Schistosomiasis

The biological cycle of the parasite consists of two phases: The phase of the definitive host (vertebrate/man) and the phase of the intermediate host (mollusk). There are two larva passages of free life in the aquatic environment that alternate with parasitic phases. Adult worms live in blood vessels. In the case of *S. mansoni* and *S. japonicum*, they can attach to the intestine or the liver of the vertebrate host. Egg laying occurs in intestinal capillary vessels where they are directed into the intestinal lumen and exit into feces. Upon contact with water, the eggs will swell, hatch, and release ciliated larvae (miracidia) that can penetrate soft parts and develop the intermediate cycle upon finding snails. They can then generate sporocysts and cercariae later. Cercariae are the second parasite free life form. Finally, when cercariae find the skin of a vertebrate host, they become schistosomula and finally migrate to the liver where they become adults, completing the life cycle [22].

In liver infections, hepatic granuloma and periportal liver fibrosis are the most important pathogenic events in schistosomiasis and are mediated by several lymphocyte subpopulations, inducing inflammatory and fibrotic response around eggs housed in different tissues. At the time of oviposition, about 60% of eggs will reach the intestinal lumen. The rest will be destroyed in capillaries of the intestinal mucosa. Some eggs will remain there, while others will be carried by the mesenteric circulation to the liver where they will reside in hepatic sinusoids. The release of soluble antigens from eggs can induce the mobilization of macrophages, eosinophils, lymphocytes, and plasma cells. Macrophages are placed in contact with the egg, forming syncytial multinucleated masses. Some will differentiate into fibroblasts with an extensive production of collagen. Moreover, by migrating to the lungs and liver, schistosomula can cause arteriolitis, arteritis, and necrosis in addition to acute hepatitis with infiltration of neutrophils, lymphocytes, and eosinophils [23,24]. In general, the formation of granulomas in disparate organs and tissues explains manifestations of the disease, including portal hypertension, the formation of pseudotumors, neurological dysfunctions, and pulmonary vascular lesions. Another pathophysiological mechanism of relevance is the occurrence of an antigen-antibody reaction that may occur at high levels with the formation of circulating immunocomplexes that can be deposited in renal vessels, causing schistosomal nephropathy [24].

On the other hand, adult worms of *S. haematobium* species live within the urogenital venules, where they digest erythrocytes. The bladder, lower ureters, urethra, seminal vesicles, cervix, uterus, and vagina are most commonly affected. Unlike other schistosomes that live within the mesenteric venules and release their eggs into the host’s intestines, *S. haematobium* releases its eggs into the urinary tract. The eggs can be eliminated by the urine or remain installed in the urogenital mucosa, causing polyps, nodules, and “sandy patches”, a calcified schistosome ova within atrophied mucosa that seems like sand in cystocopy and colposcopy. Moreover, the progression of the urogenital infection can lead to fibrosis and calcification of the bladder wall, causing obstruction, bacteriuria, and bladder cancer [4].

The pathogenesis of schistosomiasis is derived from the host-parasite interaction. The strain, the evolutionary phase, the intensity, and the number of infections of *Schistosoma* are all important factors in its associated pathology [25]. On the other hand, the host organism response can vary according to genomic constitution, the predominantly injured organ, recidivate, food pattern, ethnicity, pharmacological treatment, associated infections, in utero sensitization and, above all, the immune profile before, during, and after infection. From the first 12 h after penetration of cercariae, an important dermal and subdermal inflammatory reaction is observed. This inflammatory reaction is predominantly caused by mononuclear and polymorphonuclear cells, leading to symptoms such as a pruritic maculopapular rash [25,26].

## 3. The Use of Drugs in the Treatment of Schistosomiasis

Pharmacotherapy is the most effective method for reducing the number of infection cases of schistosomiasis. In the last four decades, access to more effective drugs has reduced the prevalence and morbidity of this disease in various countries [4,27]. In the therapeutic treatment for an acute form of schistosomiasis, the use of prednisone, a corticosteroid, or its association with oxamniquine or praziquantel, is recommended. For the treatment of chronic disease without advanced lesions, praziquantel and oxamniquine are mostly indicated. In patients with involvement of the spinal cord (schistosomal myelopathy), the use of schistosomicides and steroids has been shown to be effective in most cases. At this rate, corticosteroids should be maintained for several months after clinical improvement and they should be withdrawn slowly. Such combination is also the therapy of choice for individuals with an advanced hepatosplenic form presenting portal or pulmonary hypertension who may develop hepatitis or pneumonitis due to the embolism of dead worms after treatment [28].

Mechanisms of action of the two main antiparasitics (praziquantel and oxamniquine) are very different. Praziquantel probably interferes with the muscular activity of the parasite, causing paralysis and preventing its binding with the host tissue. It also causes electrolyte imbalance and leads to destruction of the schistosome [29]. Oxamniquine acts by its anticholinergic effect. It can irreversibly inhibit enzymes that synthesize nucleic acids [30,31].

Despite the historical use of these schistosomicides, side effects such as metallic taste in the mouth, abdominal pain, diarrhea, asthenia, headache, dizziness, decreased therapeutic efficacy, and resistance have been reported [28]. Furthermore, the use of corticosteroids can culminate in the development of several side effects of this pharmacological class, such as Cushing’s syndrome, metabolic dysregulation, and immunosuppression [32]. Other drugs used for this purpose also have many side effects with broad parasitic resistance and/or poor efficacy and some of them have been discontinued, such as metrifonate, an organophosphate with good efficacy against *S. haematobium*, but it caused abdominal pain, nausea, vomiting, diarrhea, headache, and vertigo [33]. On the other hand, artemisinine derivatives useful in the treatment of schistosomiasis and other parasitic diseases such as fasciolosis and triphostomy have been gradually neglected due to the increasing resistance in individuals co-infected with the malaria parasite [34], although these drugs can act synergistically with the heme group, generating free radicals that are toxic to the schistosomulae and are highly active against juvenile worms, whereas praziquantel is active against adult forms [35].

## 4. Biological Control of Intermediate Hosts as an Alternative Way

The control of the malacological population is an alternative way to prevent parasitic diseases that have mollusks in the life cycle of the parasite. The practice of combating natural breeding sites of intermediate hosts through the use of molluscicides has been one of alternative ways to decrease the incidence of some diseases such as schistosomiasis. However, most of these molluscicides have disadvantages such as damage to the ecosystem. Thus, there has been increasing interest in searching for new molluscicides from natural products that are less harmful to the environment [36].

Clinical treatment, prophylaxis, and prevention are performed in countries susceptible to schistosomiasis. In many cases, prevention methods such as reducing *Schistosoma* intermediate hosts (*Biomphalaria* and *Oncomelania*, for example) are important in controlling the disease, when combined with other strategies, such as the improvement of water sanitation, education, and access to clean water. However, vectors have developed resistance to chemical substances that are commonly used to inhibit the development and propagation of snails, leading to the search for new drugs and substances to be used in snail control [20].

Metallic salts such as copper sulphate were the first molluscicidal agents used. However, over time, they caused serious ecological imbalance since they limited the growth of algae that served as food for fish [3]. With the development of new molluscicides such as nicotinanilide, organotin, dibromo-nitrobenzene, sodium pentachlorophenate, tritylmorpholine, acetamide, and niclosamide, the environmental imbalance problem has become less drastic [37]. However, resistance in mollusks, residual toxicity, and low selectivity of these agents continue to be important reasons for the search and development of more selective, safe, and effective molluscicides [36].

Niclosamide is a drug of choice for controlling mollusks involved in the schistosomiasis cycle. It is also used as anthelmintic in humans [38]. This substance acts by inhibiting the anaerobic phosphorylation of adenosine diphosphate (ADP) by mollusk mitochondria, thus blocking the process of obtaining energy dependent on the fixation of CO_2_ [39]. In addition to the problems mentioned above, niclosamide is costly and easily degraded under sunlight [40].

In recent years, the aim to control and/or prevent schistosomiasis has been listed as a top priority in the agenda of the government, pharmaceutical companies, and international agencies triggered by initiatives derived from the World Health Agency resolutions [41]. The WHO program targets deworming of at least 75% of school children. However, only 28% of this figure was met in 2015 [42] mainly due to the scarce access to praziquantel and prophylactic chemotherapy. In addition, being the sole drug marketed for schistosomiasis therapy, there are some evidence points that praziquantel might be inefficacious against distinct developing strains of the parasite [43,44,45]. Other instances have demonstrated that intensive use of praziquantel can result in declined cure rates, higher resistance, and ultimately treatment failure [46]. The rising cost of praziquantel has also contributed to less successful prophylactic programs since most prevention programs depend on the availability of charitable funding organizations. In addition, in many countries, the control of schistosomiasis is not integral of the national budget, meaning that resources are limited to overcome the morbidity and mortality associated with the disease [41,42,47]. Given the limitation in available resources and the fact that there is no available vaccine for this disease, there is a dire need to develop alternative medicines that are both cost-effective and treatment-effective for this disease [48,49]. In this advent, natural products present an interesting opportunity toward the development of novel pharmacological agents to triumph over this disease.

The prevalence of schistosomiasis is largely dependent on the intermediate host ecology. Controlling the intermediate host undeniably can control the disease [50]. In this advent, plants with proven molluscicide activities are cheap and environmentally friendly for the control of parasite. Since the past century, several studies have focused on molluscicidal activities of endemic plants from the most affected regions in the world. Plant candidates include the following: *Apodytes dimidiata* E.Mey.ex Arn., *Ambrosia maritima* L., *Anacardium occidentale* L., *Croton macrostachys* L. Hochst.ex A.Rich., *Phytolacca dodecandra* L’Herit, *Swartzia madagascariensis* Desv., *Phytolacca dodecandra* L’Hér., *Sapindus Saponaria* L., *Swartzia madagascariensis* Desv., *Berkheya speciosa* (DC.) O.Hoffm., *Balanites maughamii* Sprague, *Warburgia salutaris* (G.Bertol.) Chiov., *Combretum imberbe* Wawra, *Combretum molle* R.Br. ex G.Don, *Euclea natalensis* A.DC., *Apodytes dimidiata* E.Mey. ex Arn., *Gardenia thunbergia* Thunb., and *Solanum nodiflorum* Jacq. [51,52,53,54,55].

On the other hand, several plants have the potential to interfere with the life cycle of worms, thereby impeding their growth, locomotion, and ability to lay eggs. A plethora of such plants have been highlighted by the scientific community, including *Jatropha elliptica* (Pohl) Oken, *Asparagus stipularis* Forssk, *Sanguinaria canadensis* L., *Curcuma longa* L., *Plectranthus neochilus* Schltr., *Hemerocallis fulva* (L.) L., *Schefflera vinosa* (Cham. and Schltdl.) Frodin and Fiaschi, *Cleome droserifolia* (Forssk.) Delile, *Clerodendrum umbellatum* Poir., *Artemisia annua* L., *Baccharis trimera* (Less.) DC., *Persea americana* Mill., *Allium sativum* L. [56], *Abrus precatorius* L., *Acacia karroo* Hayne, *Maytenus senegalensis* (Lam.) Exell, *Peltophorum africanum* Sond., and *Ziziphus mucronata* Willd. [57]

## 5. Recent Studies on Natural Resources of Affected Regions as an Alternative in the Treatment and Control of Schistosomiasis

Within the context mentioned above, the search for new and effective drugs with fewer side effects is necessary. Hence, the active agents of vegetal origin that are abundant in number and diversity in many affected countries could be a promising alternative. According to Ali (2011), several plant species have been used in different regions of the world for the treatment of parasitic diseases, including *Allium sativum*, *Chenopodium ambrosoides*, *Curcubita pepo*, *Olea europaea*, *Mentha crispa*, *Citrus reticulata*, and phytopharmaceuticals such as berberine and lapachol [33,58]. However, an increasing number of studies have demonstrated the importance of endemic plant species and their derivatives with a specific action against the genus *Schistosoma* or against one of their intermediate hosts. Such endemic plant species and its derivatives can serve as prophylactic and/or curative agents in countries where they are found. The objective of this review is to summarize recent studies (since 2010) on endemic species, especially species from the most affected countries. In the following sections, we describe the most promising plant derivatives according to their native region.

### 5.1. Brazil

Brazil is one of the most affected countries by schistosomiasis, presenting 12,009 new cases in 2016 and about 1.5 million people are at risk of contracting this disease. *Schistosoma mansoni*, the main etiologic agent of this disease in Brazil, can be transmitted to the population through three species of mollusks: *Biomphalaria glabrata*, *B. straminea*, and *B. tenagophila* [59]. This country has a vast area of equatorial, tropical, and subtropical climate, so that it is suitable for the development of different strains of snails, as well as the parasite’s cycle. On the other hand, the country also has the greatest biodiversity on the planet, which potentially has a large number of plant resources to be researched in the combat against schistosomiasis [60].

In 2010, Parreira et al. [61] described the activity of essential oil (10, 50, and 100 μg/mL) from leaves of *Baccharis dracunculifolia*, a species used by folk medicine, against *Schistosoma mansoni* adult worms. After the treatment, worms showed a significant decrease in their motor activities. In addition, most pairs of coupled adult worms were separated into individual males and females. Furthermore, adult worms of *S. mansoni* showed integumentary alterations after treatment with the essential oil. Their study also demonstrated that the essential oil had no toxicity against VERO cells. Furthermore, it showed that nerolidol and spathulenol were major substances in the essential oil [61]. In the same year, Magalhães et al. [62]. showed that the action of fluoroglucinol derivatives isolated from species from genus *Dryopteris* is known to have a global distribution against *S. mansoni.* Aspidin at 25 to 100 μM, flavaspidic acid at 50 and 100 μM, methylene-bisaspidinol at 100 μM, and desaspidin at 25 to 100 μM were the most active derivatives, causing death of adult worms. These fluoroglucionols at 100 μM also inhibited egg development. Furthermore, the authors suggested that schistosomicidal effects of phloroglucinols derivatives might be related to the inhibition of oxidative phosphorylation pathway in *S. mansoni* [62].

*Ageratum conyzoides* L. (Asteraceae), an annual aromatic weed from Southeastern Brazil, can produce leaf essential oil rich in Precocene I and (*E*)-caryophyllene [63]. The synergic effect of main compounds against adult worms of *S. mansoni* has been reported. *Plectranthus neochilus*, another Brazilian southeastern plant, has also been evaluated for its activity against *S. mansoni*. The essential oil of its leaves at a concentration of 100 ppm caused 100% mortality in a period of 24 h. Furthermore, it caused separation of coupled pairs, decrease of motor activity, and tegumental alterations. The main substances of its essential oil were β-caryophyllene, α-thujene, and α-pinene [63]. In the same year, another group of researchers also demonstrated the antiparasitic activity of cramoll-1,4-lectine isolated from seeds of *Cratylia mollis*, an endemic species from the Brazilian northeast [64]. Treatment with this substance at 50 mg/kg for 40 days or at 7 mg/kg for seven days reduced egg excretion (79% or 80%), adult worm recovery (71% or 79%), and liver granulomas (40% or 73.5%) caused by *S. mansoni* in infected mice [64].

In vitro activity of piplartine, an amide isolated from inflorescences of *Piper tuberculatum* found in Brazil, has been reported [65]. Piplartine at a concentration of 15.81 μM reduced the motor activity of worms of *S. mansoni* and caused their death within 24 h. In addition, this substance induced morphological changes on the tegument of adult worms. A quantitative analysis revealed an extensive tegumental destruction, represented by the number of damaged tubercles. In addition, this amide was not toxic to the VERO cells when it was used at concentrations up to three times higher than the one showing schistosomicidal effects (31.51 μM) [65]. Another study demonstrated that imidazole alkaloid epiisopiloturine had an anti-*S. mansoni* activity. This substance was found in leaves of *Pilocarpus microphyllus*, a native plant species of Amazonia and Brazilian savannah. This alkaloid has been shown to be active against parasites of different developmental stages, including adults, schistosomulas, and eggs. Epiisopiloturine at a concentration of 300 μg/mL caused the death of all schistosomula within 120 h. Extensive tegumental alterations and death were observed when adult schistosomes were exposed to 150 μg/mL of epiisopiloturine. At the highest sub-lethal dose (100 μg/mL), a 100% reduction in egg laying of paired adult worms was observed. Furthermore, it exhibited no cytotoxicity to mammalian cells [66].

Oliveira et al. [67] have investigated the activity of *Baccharis trimera* against *S. mansoni*. This species is commonly used as a phytotherapic agent in Brazilian traditional medicine due to its several pharmacologic activities. They found a significant decline in the motility of worms with a mortality rate of 100% at 30 h after exposition to the leaf essential oil at a concentration of 130 μg/mL. Male worms were more susceptible, producing a dose-response effect within a shorter exposition period than female worms. The essential oil of *B. trimera* also induced a peeling on the tegument surface as well as destruction of tubercles and spines, resulting in smooth areas on the body surface. The essential oil also caused tegument destruction in female worms. In addition, it caused destruction of oral and acetabular suckers. Moreover, the cytocidal effect was only observed at the highest concentration (250 μg/mL), indicating a low cytotoxicity of this essential oil [67].

Miranda et al. [68] have investigated the antiparasitic action of steroidal alkaloids from *Solanum lypocarpum*, a Brazilian medicinal plant known as “wolf fruit”. In vitro schistosomicidal activities isolated from steroidal alkaloids were evaluated against adult worms. The alkaloidic extract (20, 32, and 50 μg/mL), solasonine (50 μM), solamargine (32 and 50 μM), and equimolar mixture of glycoalkaloids (20, 32, and 50 μM) caused the separation of all coupled worms and extensive disruption on their teguments such as sloughing. It also caused death within 24 h of incubation. In addition, the alkaloidic extract (10 and 15 μg/mL), solasonine (50 μM), solamargine (10, 15, and 20 μM), and equimolar mixtures of glycoalkaloids (10 and 15 μM) reduced the development of eggs produced by adult worms. There was a synergistic effect between solamargine and solasonine [68].

In 2014, Brazilian researchers evaluated the molluscicidal activity of *Schinopsis brasiliensis* against *Biomphalaria glabrata*. Extracts in chloroform and ethyl acetate from the stem bark caused mortality of *B. glabrata*, with IC_90_ values of 68 and 73 µg/mL, respectively [59]. In 2018, Faria et al. investigated effects of plants on the control of *B. glabrata*. *Manilkara subsericea*, an endemic plant to the Brazilian sandbanks of Rio de Janeiro State, has a wide range of biological activities. At a concentration of 250 ppm, the *M. subsericea* leaf crude extract and ethyl acetate fraction induced 80 ± 4.13% and 86.66 ± 4.59% mortalities of adult snails after 96 h of exposure. LD_50_ values were 118.7 ± 1.62 and 23.41 ± 1.15 ppm, respectively. Substances isolated from *M. subsericea* were also found to be active in controlling *B. glabrata*. Treatment with quercetin, myricetin, and ursolic acid at a concentration of 100 ppm for 96 h induced mortalities of 100%, 80%, and 53.33%, respectively [69]. One year later, another study reported the activity of a Brazilian sandbank species against *B. glabrata* [70]. Nanoemulsified essential oil from leaves of *Xylopia ochrantha*, an Annonaceae plant, showed activity against three species of *Biomphalaria*: *B. glabrata*, *B. tenagophila*, and *B. straminea*. The similar action was observed on mollusks of different ages and its oviposition. Treatment with this essential oil at a concentration of 78 ppm for 24 h caused 100% mortality of all adult species [70]. Table 2 shows recent studies with Brazilian plants with antischistosoma or molluscicide activity.

### 5.2. Africa

In Africa, schistosomiasis is endemic in rural and coastal regions. Its geographical conditions are suitable for infestation of this parasite due to multiple creeks, creeklets, lakes, ponds, rivers, and almost stagnant water sources [73]. Being a neglected tropical disease, schistosomiasis strikes mostly poor, marginalized communities where people are in contact with natural water sources, posing an enormous societal, healthcare, and economic burden, mostly in Sub-Saharan Africa (SSA) [74,75]. Furthermore, this disease has a profound adverse effect on maternity, the development of children, and agricultural output. Schistosomiasis remains one of the prime factors contributing to poverty among 500 million SSA residents. It has been estimated that around 120 million individuals were present with schistosomiasis-related symptoms in Sub-Saharan Africa. This constitutes around 85% of the Sub-Saharan Africa population which represents 13% of the world’s populace [76]. In addition, 20 million individuals from the same region undergo an incredible torment due to chronic manifestations of this disease which ranks the second right after hookworm infection in SSA. Among African countries with the highest prevalence of schistosomiasis, Nigeria has the highest number (29 million) of cases, followed by the United Republic of Tanzania, Ghana, and the democratic republic of Congo with 19 and 15 million cases each [17,34]. Nonetheless, it is believed that most cases of the disease are not reported. The true incidence of this disease might be 400–600 million cases worldwide [34].

#### 5.2.1. Plants Traditionally Used against Schistosomiasis

A plethora of plant species have been employed traditionally against schistosomiasis in SSA. They have been recorded in both early and recent ethnobotanical surveys conducted in Africa. These plant species include *Abrus precatorius* L. subsp. africanus Verdc., *Afzelia quanzensis* Welw., *Antidesma venosum* E. Mey. ex Tul., *Boswellia carteri* Birdw., *Berkheya speciosa* (DC.) O.Hoffm., *Cassia abbreviata* Oliver subsp., *Cissampelos murconata* A. Rich. *Euclea divinorum* Hiern., *Euclea natalensis* A. DC., *Faurea saligna* Harv., *Macaranga kilimandscharica* Pax., *Maytenus senegalensis* (Lam.) Excell., *Mondia whitei* Skeels, *Ocimum americanum* L., *Ocimum canum* Sims, *Ormocarpum trichocarpum* (Taub.) Engl., *Pterocarpus angolensis* DC., *Protasparagus buchananii* (Baker) Oberm., *Rhus gueinzii* Sond, *Rumex lanceolatus* Thunb., *Rumex nepalensis* Spreng., *Sclerocarya birrea* (A. Rich.) Hochst. subsp. caffra, *Tephrosia macropoda* Harv., *Terminalia phanerophlebia* Engl. and Diels, *Vernonia amygdalina*, *Ximenia americana* L. var. americana, and *Ximenia caffra* Sond [77,78,79,80,81].

Among the plant species traditionally employed against *Schistosoma mansoni*, only a few have been subjected to scientific screening (Table 3). Principally, in vitro assays have been performed on these plant species and the most potent result was retrieved for the root extract of *Abrus precatorius* L. subsp. africanus Verdc. which nonetheless deserved to be evaluated further in vivo and in randomized clinical trials.

#### 5.2.2. Recent Scientific Studies on Molluscicide Activity

In 2010, Adetunji and Salawu [84] reported that *Terminalia catappa* and *Carica papaya* possessed molluscicide activity against *Bulinus globosus* and *Biomphalaria pfeifferi*, respectively, the main intermediate hosts of *S. haematobium* and *S. mansoni*. Despite the fact that both vegetal species are distributed in several tropical regions of the world, they also have good adaptation in Nigeria and affect mollusks and parasites of great importance in this country. In that study, an ethanolic extract of *T. catappa* leaf showed LC_50_ values of 864.1 and 2716.3 ppm against *B. pfeifferi* and *B. globosus*, respectively. However, an ethanolic extract of *C. papaya* leaf showed LC_50_ values of 1095.7 and 619.1 ppm against *B. pfeifferi* and *B. globosus*, respectively [84]. One year later, Nigerian scientists described activities of several extracts of leaves and fruits of *Blighia unijugata*, an endemic plant in tropical Africa, against *B. glabrata*. LC_50_ values of these extracts were found to be 7.60 and 13.00 μg/mL. Among various extracts, the ethyl acetate extract of its fruit was the most active one [57]. Part of these authors also evaluated the activity of *Zanha goluogensis* against the same mollusk. It was found that the ethanolic extract of its stem showed an LC_50_ value of 60 ppm [82].

In 2013, Angaye [85] investigated the activity of different extracts of *Jatropha curcas* against mollusks *Bulinus globosus*, *Bulinus rholfsi*, and *Biomphalaria pfeifferi*. It was found that the methanolic extract of its leaves was active against all species, showing LC_50_ values of 0.3 and 30 ppm against *Bulinus* and *B. pfeifferi*, respectively, while the crude ethanolic extract showed less activity (LC_50_ > 500 ppm) [85,86]. Previously, other authors have also reported activities of root and seed methanolic extracts against different species of *Bulinus* [87,88]. Two years later, Angaye et al. [89] reported activities of two typical mangrove species, *Avicennia germinans* and *Rizhophora mangle*, from Niger Delta. For the first species, the methanolic leaf extract was active against *Biomphalaria pfeifferi*, *Bulinus globosus*, and *Bulinus rholfsi*, showing LC_50_ values of 175, 89.21 and 123.74 ppm, respectively, whereas the methanolic leaf extract of *R. mangle* showed LC_50_ values of 87.50 and 108.22 ppm for the two first mollusks and *B. rholfsi*, respectively [89]. Aqueous and ethanolic extracts of *Vernonia amygdalina* have been found to be toxic to adult *Biomphalaria pfeifferi*, showing an LC_50_ value of 338.8 ppm and an LC_90_ value of 614.8 ppm [90]. Interestingly, previous studies with this species have reported its antischistosomal activity [91,92,93] and its use in etnomedicine as a folkloric treatment against haematuria resulting from *Schistosoma haematobium* infection and stomach complaints due to infestation by *Schistosoma mansoni* [51]. Studies of African plant species and their molluscicidal activity are summarized in Table 4.

#### 5.2.3. In Vitro Investigation of Medicinal Plants against Schistosoma

One of the most extensive studies about plant schistosomicidal activity was conducted by Yousif et al. in 2011 [98]. These authors screened 281 Egyptian plants, of which 14 showed high activities against *S. mansoni* adult worms after treatment for 24 h: *Callistemon viminalis* (Soland. Ex Gaertn) Cheel, *C. rigidus* R. Br., *C. speciosus* (Sims.) DC, *C. citrinus* Stapf. *Eucalyptus citriodora* Hook, *Eucalyptus rostrata* Dehnh. *Eugenia edulis* Vell, *E. javanica* Lam, *Melaleuca leucadendron* (L.) L. *M. stypheloides* Sm (all belong to Myrtaceae)*, Cryptostegia grandiflora* R. Br. (Asclepiadaceae)*, Zilla spinosa* (L.) Prantl (Cruciferae), *Ficus trijuja* L.(Moraceae), and *Fagonia mollis* Delile (Zygophylacae) (Table 5) [98]. Moreover, in Egypt, another study on asparagaline A, a triterpenoid isolated from roots of an African plant *Asparagus stipularis* Forss., demonstrated its activity against *S. mansoni* [99]. The administration of asparagalin A resulted in a retardation of worm growth and locomotion on the first day. It also showed a significant activity of egg-laying suppression at a 200 µg/mL concentration [99]. In this same year, Ramalhete et al. [100] reported that triterpenes isolated from the methanol extract of aerial parts of *Momordica balsamina* L. had a potent in vitro schistosomicidal potential (100% within 24 h). Balsaminol F and karavilagenin C showed LC_50_ values of 14.7 ± 1.5 and 28.9 ± 1.8 µM, respectively, after 24 h of incubation. Both compounds at 10–50 µM induced significant reductions in the motor activity of worms and significantly decreased egg production. Furthermore, at 10-100 µM, they were able to separate adult worm pairs into males and females after 24 h [100].

In 2015, a study investigated the effect of an aqueous extract (1.25–40 mg/mL) of aerial parts of *Sida pilosa* Retz. (Malvaceae) and derived n-hexane, dichloromethane, ethyl acetate, and n-butanol fractions (0.25–8 mg/mL) against *Schistosoma mansoni*. Among these extracts, the n-butanol fraction was the most active one, showing an LC_50_ value of 1.25 mg/mL [45]. Two years later, Tekwu et al. [101] demonstrated that the stem bark and root extracts of *Rauwolfia vomitoria* at concentrations of 250–1000 µg/mL were active against *Schistosoma mansoni* worms after 120 h of incubation. In addition, the cytotoxicity (MTT) assay conducted on HepG2 and Chang liver cells demonstrated that these extracts were safe. They inhibited the proliferation of these cell lines with an IC_50_ > 20 µg/mL [101].

### 5.3. Asia

#### 5.3.1. Prevalence of Schistosomiasis in Asia

In Asia, the major foci of human schistosomiasis infection caused by parasite *Schistosoma japonicum* are in China, the Philippines, and small pockets of Indonesia. To a lesser extent, along the Mekong river on borders of Cambodia and Laos People’s Democratic Republic, the infection is caused by *S. mekongi* [102,103]. *S. japonicum* and *S. mekongi* are traditionally considered as zoonotic [104]. Specific intermediate snail hosts for *S. japonicum* and *S. mekongi* are *Oncomelania hupensis* and *Neotricula aperta*, respectively [102,105]. Approximately 600 million people are estimated to be at risk of infection in China. Approximately 0.3 million people are currently infected. In the Philippines, 6.7 million people live in endemic areas. Of these, 1.8 million people are considered to be directly exposed to infection through water contact activities [106,107]. Approximately 140,000 people are estimated to be at risk for *S. mekongi* infection (80,000 people in Cambodia and 60,000 in Lao PDR) [102,108].

In China, schistosomiasis japonica remains a major public health concern. It is listed as one of the top priorities in communicable disease control defined by the central government [109,110]. The major endemic foci are concentrated on marshland and lake regions of Southern China which cover a vast area of five provinces (Jiangsu, Anhui, Hubei, Jiangxi, and Hunan). Cases within the area account for 86% of the total number of people infected in China [103,111]. It should be noted that the schistosomiasis transmission period in China lasts only for five months annually over two distinct transmission periods, whereas in the Philippines it is throughout the year [103,112]. For *S. mekongi*, its transmission period is March to April, coinciding with the dry season when the water level is low and host snail populations reach their maximum [102]. In Japan, eradication of the disease was achieved through transmission control by environmental management (i.e., land reclamation to enhance agricultural production and cementing ditches used for rice irrigation) and social economic development [103,113]. China has used extensive, long-term, repeated praziquantel chemotherapy to control the morbidity and reduce the prevalence and intensity of the *S. japonicum* infection in the country over three decades. Although *S. japonicum* has not shown resistance to praziquantel yet [114], the emergence of drug resistance has been experimentally induced in the laboratory, proving that *S. japonicum* may develop resistance to praziquantel under drug selection pressure [115,116]. As the potential development of praziquantel resistance may pose a great threat to the elimination of *S. japonica* in China and other Asian countries, there is an urgent need to find antischistosomal agents, especially from natural sources that are generally safer.

#### 5.3.2. Plants Traditionally Used against Schistosomiasis in Asian Countries

*Ginkgo biloba* is a Chinese-specific rare relict species with relatively high economic and medicinal values. Its sarcotesta is usually discarded. However, it contains high levels of ginkgolic acids. Ginkgolic acids are long-chain phenolic compounds that are derivatives of sumac acid. Reports have shown that ginkgolic acids possess biological activities, including anti-tumor, neuroprotective, anxiolytic, and antibacterial activities [117,118,119,120]. Considering such biological virtues of *G. biloba*, Li et al. [121] have evaluated the petroleum ether fraction of the ethanolic extract of fallen leaf of *G. biloba* (PFGB) against *O. hupensis*. This extract showed a high toxicity (100% mortality) after treatment at a concentration of 100 mg/L for 72 h. Out of five fractions of the ethanolic extract of *G. biloba* tested for molluscicidal activity, the petroleum ether fraction (PFGB) exerted the most pronounced activity in a time- and dose- dependent manner. Experimental results demonstrated that the LC_50_ value of PFGB was decreased from 72.38 mg/L at 24 h to 9.22 mg/L at 72 h. Upon assessment, it was found that the glycogen and total protein contents of the snail’s tissues decreased at a rate parallel to molluscicidal activity. Therefore, abnormal energy metabolism might be a factor contributing to its molluscicidal activity [121].

*Buddleja lindleyana* is a medicinal plant distributed in Eastern China, including Jiangsu, Anhui, Jiangxi, and Hubei provinces. This plant has been traditionally used for treating various ailments such as rheumatism, cough, and blood stasis [122]. Chemical investigations of *B. lindleyana* have revealed compounds such as phenethyl alcohol glycosides, phenylpropanoid phenolic glycosides, sesquiterpenes, diterpenes, triterpenes, flavonoids, and other constituents. One study has revealed that the N-butanol fraction of *B. lindleyana* (NFBL) is potent against the snail *O. hupensis* (LC_50_ = 39.1 ppm, LC_90_ = 59.3 ppm) [120]. Han et al. [123] have further reported that active components of *B. lindleyana* can induce remarkable decreases of activities of five key enzymes: Succinate dehydrogenase (SDH), lactate dehydrogenase (LDH), cytochrome oxidase (CC0), cholinesterase (CHE), and nitric oxide synthase (NOS). These decreased activities of enzymes affected the supply of neurotransmitters (CHE and NOS) and energy supply (CCO, LDH, SDH), leading to a physiological function disorder or loss which ultimately resulted in the death of snails [123]. A further study has revealed that acacetin-7-rutinoside is the molluscicidal component of NFBL. In addition, acacetin-7-rutinoside at a concentration of 100 mg/L was found to be non-toxic to zebrafish, implying that this active compound might have applications as a potent molluscicide against *O. hupensis* [124].

*Pulsatilla chinensis* (Bunge) Regel (PRS) is a botanical with a long history in medical use in China. It displays “blood-cooling” and detoxification activities. Roots of *Pulsatilla chinensis* (Bunge) Regel have been widely used for treating various ailments such as intestinal amebiasis, malaria, trichomoniasis, bacterial infections, and malignant tumor [125]. A study carried by Chen et al. in 2012 showed that PRS displayed similar molluscicidal activity against *O. hupensis* (LC_50_ at 24 h: 0.48 mg/L) to niclosamide as a positive control (LC_50_ at 24 h: 0.16 mg/L). Effects of PRS on cholinesterase (CHE), alanine aminotransferase (ALT), alkaline phosphatase (ALP), and lactate dehydrogenase (LDH) activities in cephalopodium and liver of snails were assessed. It was found that there were significant alterations in CHE, ALP, and ALT activities in the cephalopodium and the liver of snails after exposure to 40% and 80% LC_50_ of PRS or NIC for 24 h. Furthermore, the zebra fish lethality test was performed to assess its toxicity to non-target aquatic species. Results showed that PRS which contained 15 compounds was less toxic to zebra fish than the control (niclosamide) [126]. In 2018, Kang et al. investigated whether hederacochiside C (HSC) isolated from roots of *P. chinensis* possessed antischistosomal effects and anti-inflammatory activities in *S. japonicum*-infected mice. Mice infected by schistosomula or adult worms by an intravenous injection were treated with different concentrations of HSC twice a day for five consecutive days. Experimental results demonstrated that the total worm burden, female worm burden, and egg burden in livers of mice treated with 400 mg/kg HSC were fewer than those in non-treated ones. Following the HSC treatment, murine immune responses were assessed by enzyme-linked immunosorbent assays (ELISA). Results showed that 200 mg/kg of HSC was sufficient to reduce the expression of IgG, tumor necrosis factor (TNF)-α, interleukin (IL)-4, and IL-17 in comparison with those in the infected group, exhibiting remarkable immunomodulatory effects [127]. More recently, Kang et al. reported antischistosomal properties of hederacolchiside A1 (HSA) isolated from *Pulsatilla chinensis* against *S. japonicum* and *S. mansoni*. Its antischistosomal activity was higher than praziquantel and artesunate against one-day-old juvenile schistosome. In vivo assays confirmed that the HSA-mediated antischistosomal activity was partly due to morphological changes in the tegument system when juvenile schistosomes were exposed to HAS [127]. Extensive tegumental disruption such as sloughing and erosion was observed. The tegument of schistosome is a protective sheath that plays a crucial role in host-parasite interactions as well as in defense, uptake of nutrients, osmoregulation, and excretion and hence morphological changes in the tegument is critical for the survival of schistosome [56,128].

Reports have highlighted the molluscicidal activity of the leaf extract *from C. camphora* against *O. hupensis* [129,130]. Yang et al. [131] have investigated molluscicidal and larvicidal activities of leaf extracts of *C. camphora* growing in China against *O. hupensis* and *S. japonicium*. Gas chromatography coupled to mass spectrometry (GC-MS) was performed to identify bioactive components from the leaf extract of *C. camphora*. Results showed that Linalool-rich *C. camphora* leaf extracts had high molluscicidal effects against *O. hupensis* (LC_50_ = 0.25 mg/L) and cercaricidal activity against *S. japonicium* (LC_50_ = 0.07 mg/L) [131].

Xiao et al. [132] have evaluated schistosomicidal activities of flavonoids isolated from *Astragalus englerianus*, a traditional Chinese medicine plant. Among the isolated flavonoids, 2,2′,5′-trihydroxy-4-methoxychalcone and (3R)-sativan caused 100% mortality of worms within 12 h after treatment with a RPMI 1640 medium containing each drug (0.70 and 0.77 mM, respectively) [132].

Wan et al. [133] evaluated cercaricidal activities of *Allium sativum* (garlic) oil against *S. japonicum* larvae both in vitro and in vivo. Their findings revealed that exposure to garlic emulsions at concentrations of 10^−6^ (*v*/*v*) or higher for 30 min induced a 100% mortality of *S. japonicum* cercariae. The toxicity of the garlic oil against *S. japonicum* was determined by pre-treating mice with garlic emulsion on the shaved abdomen, followed by an *S. japonicum* cercariae challenge. The in vivo assay revealed that the pre-exposure treatment with ≥ 10^−4^ (*v*/*v*) garlic emulsions in mice caused a 100% inhibition of *S. japonicum* infection in mice, while the pre-treatment with 10^−5^ and 10^−6^ (*v*/*v*) emulsions achieved 20–40% inhibition of *S. japonicum* infection and 35.2% to 63.6% worm burden reduction, respectively [133]. Similarly, curcumin, a major polyphenol isolated from rhizomes of *Curcuma longa* L., a dietary spice widely used in Asian cuisine and in folk medicines worldwide, has been reported to be active in vitro against every life stage of *S. japonicum* [134]. Furthermore, curcumin was observed to exert an optimal activity against the adult stage without differential sensitivity between male and female worms. After 72 h of incubation with 5 mg/mL of curcumin, a decrease in the motor activity of these worms without tegumental alterations was observed by Ke et al. [134].

*Macleaya cordata* (Willd) R. Br. is a plant with high content of alkaloids. The molluscicidal effect of alkaloid components against snail *Oncomelania hupensis* was determined by Ke et al. [135]. Alkaloid AN2 was found to be the most toxic one against snail *O. hupensis*, showing 48 h LC_50_ and LC_90_ values of 6.35 and 121.23 mg/L, respectively. Responses of some critical enzymes to AN2, including activities of alanine aminotransferase (ALT), alkaline phosphatase (ALP), malic dehydrogenase (MDH), aspartate transaminase (AST), and succinate dehydrogenase (SDH), in cephalopodium and liver were also detected. Results showed that AN2 significantly inhibited activities of MDH, SDH, and esterase isozyme. AN2 also significantly stimulated activities of ALT, ALP, and AST to increase at a low concentration (25 mg/L), while IT significantly inhibited activities of these enzymes at a high concentration (100 mg/L). These results indicate that AN2 not only can inhibit protein synthesis and respiratory chain oxidative phosphorylation, but also can cause hepatocellular injury and reduce the detoxification ability of the liver [132]. Based on the aforementioned facts and information summarized in Table 6, many Chinese herbal medicines are potential molluscicides. They can be used to control intermediate hosts of schistosome and are safer to use than their synthetic counterparts.

## 6. Conclusions

Schistosomiasis is a worldwide disease and needs the integration of several measures to promote its control, which includes clinical treatment with antiparasitic agents, as well as the use of molluscicides to promote the biological control. For these purposes, the use of plant-based actives available in the affected countries is one of the viable, ecologically friendly and cheaper alternatives, since most of them are considered developing countries. Thus, this review demonstrates that several researches with natural resources present a broad diversity of species/substances that have action in different phases of the schistosomiasis cycle and can conduct further studies with active formulations and optimization of the best candidates to be used in the combat against this disease in each country.

## Figures and Tables

**Table 1:** Summary of African regions where schistosomiasis strikes most.

Sub-Saharan African Region	Infected Individuals (Proportion)	Causal Agent	Source
Alamata district, Ethiopia	(73.9%)	*S. mansoni*	[7]
Nigeria	(56%)	*S. mansoni*, *S. mansoni* and *S. haematobium* combined infection	[11]
Sengerema district, nyamatongo ward, north-west Tanzania	School children aged 8–17 years (64.3%)	*S. mansoni*	[12]
Tono irrigation canal, north Ghana	Children aged 6–15 years (33.2%/19.8%)	*S. haematobium/S. mansoni*	[12]
Volta basin, Ghana	Adult male and female subjects (46.5%)	urinary schistosomiasis	[13]
Eastern cape province, South Africa	School-age students (73.3%)		[14]
School children, Mozambique	(47%/1%)	*S. haematobium/S. mansoni*	[15]
Zarima town, north-west Ethiopia	319 elementary school children (37.9%)	*S. mansoni*	[16]
South-west Cameroun	69.17%	*S. haematobium*	[16]

**Table 2:** Extracts, oils, and substances from the Brazilian plant origin with antischistosoma or molluscicide activities.

Plant Source	Extract/Oil/Substance	Biological Target	Ref.
*Ageratum conyzoides L.*	Leaf Essential Oil	*Schistosoma mansoni*	[71]
*Baccharis dracunculifolia*	Leaf Essential Oil	*Schistosoma mansoni*	[61]
*Baccharis trimera*	Leaf Essential Oil	*Schistosoma mansoni*	[67]
*Cratylia mollis*	Cramoll-1,4-lectine	*Schistosoma mansoni*	[64]
*Dryopteris* genus	Aspidin, Flavaspidic Acid, Methylene-bisaspidinol, Desaspidin	*Schistosoma mansoni*	[71]
*Pilocarpus microphyllus*	Epiisopiloturine	*Schistosoma mansoni*	[66]
*Piper tuberculatum*	Piplartine	*Schistosoma mansoni*	[65]
*Plectranthus neochilus*	Leaf Essential Oil	*Schistosoma mansoni*	[63]
*Solanum lypocarpum*	Fruit Alkaloidic Extract, Solasonine, Solamargine, Glycoalkaloid mixture	*Schistosoma mansoni*	[68]
*Manilkara subsericea*	Crude Ethanolic Extract, Ethyl acetate Extract, Quercetin, Myricetin, Ursolic Acid	*Biomphalaria glabrata*	[69]
*Schinopsis brasiliensis*	Stem bark chloroformic and ethyl acetate extracts	*Biomphalaria glabrata*	[72]
*Xylopia ochrantha* Mart.	Leaf Essential Oil	*Biomphalaria glabrata, B. straminea* and *B. tenagophila*	[70]

**Table 3:** In vitro studies of traditionally employed plant species against *Schistosoma mansoni.*

Plant Species	Part Used	Lethal Concentration (mg/mL) (t = 1 h)	Source
*Abrus precatorius* L. subsp. africanus Verdc.	Stem Root	1.50.6	[57]
*Berkheya speciosa* (DC.) O.Hoffm.	Aqueous plant extract	> 6.25	[82]
*Euclea divinorum* Hiern	Aqueous plant extract	50	[82]
*Euclea natalensis* A. DC.	Aqueous plant extract	> 3.13	[82]
*Maytenus senegalensis* (Lam.) Excell.	Leaves and stem Root Root and bark	25 25 2.5	[57]
*Ocimum americanum* hexane, *Ocimum americanum* water	Whole plant	Worm reduction in mice (68.7 and 63.4%) vs. praziquantel (75.2%)	[83]
*Pterocarpus angolensis* DC.	Leaves Stem Bark	102 33.8 51.3	[57]
*Sclerocarya birrea* (A. Rich.) Hochst. subsp. Caffra		> 25	[82]

t: Time; h: Hour.

**Table 4:** Scientific studies of African plant species with molluscicidal activity.

Plant Species	Plant Part /Extract	Snail Species	LC_50_	Source
*Avicennia germinans* (L.) L.	Leaf/Methanol	*Biomphalaria pfeifferi*	175	[89]
	Leaf/Methanol	*Bulinus globosus*	89.21	
	Leaf/Methanol	*Bulinus rholfsi*	123.74	
*Blighia Unijugata* Baker	fruit/ethyl acetate	*Biomphalaria glabrata*	7.60	[94]
	Pericarp/butanol		15	
	Pericarp/water		25	
*Carica papaya* L.	Leaf/ethanol	*Bulinus globosus*	619.10	[84]
		*Biomphalaria pfeifferi*	2716.30	
*Croton floribundus* Spreng	Leaf/Hexane	*Biomphalaria glabrata*	37.4	[95]
	Leaf/Methanol	*Biomphalaria glabrata*	14.8	
	Leaf/ethanol	*Biomphalaria glabrata*	4.2	
*Euphorbia helioscopia* L.	Leaf/cold water	*Bulinus wright*	80	[96]
	Leaf/hot water		96.6	
	Leaf/methanol		11.3	
	Leaf/chloroform		80.5	
	Leaf/acetone		8.9	
	Leaf/hexane		99	
*Euphorbia schimperiana* Scheele	Leaf/cold water		81.8	[96]
	Leaf/hot water		72.8	
	Leaf/ methanol		2.3	
	Leaf/ chloroform		3	
	Leaf/acetone		10.1	
*Jatropha Curcas* L.	Leaf/hexane	*Bulinus natalensis* and *Bulinus truncatus*	18	[87]
	Seed/methanol		0.25	
	Root/ethanol	*Bulinus truncatus* and *Bulinus natalensis*	60	[88]
	Leaves/methanol	*Bulinus globosus* and *Bulinus rholfsi*	0.3	[85]
	Leaves/crude extract	*Biomphalaria pfeifferi*	> 500	[86]
	Leaves/methanol	*Biomphalaria pfeifferi*	30	[86]
	Leaves/crude extract	*Biomphalaria pfeifferi*	> 500	[86]
	Seed/methanol	*Biomphalaria pfeifferi*	25	[87]
*Jatropha glauca* Vahl	Leaf/acetone	*Biomphalaria pfeifferi*	6.76	[96]
*Rhizophora mangle* L.	Leaf/chloroform	*Biomphalaria pfeifferi*	16.50	[96]
	Leaf/methanol	*Biomphalaria pfeifferi*	87.50	[89]
*Rhizophora racemosa* G. Mey.	Leaf/methanol	*Bulinus globosus*	87.50	[89]
	Leaf/methanol	*Bulinus rholfsi*	108.22	
	Leaf/methanol	*Biomphalaria pfeifferi*	150	
*Terminalia catappa* L.	Leaf/methanol	*Bulinus globosus*	125	[84]
	Leaf/methanol	*Bulinus rholfsi*	85.51	
	Leaf/ethanol	*Bulinus globosus*	1095.70	
*Tetrapleura tetraptera* (Schum. and Thonn.) Taub.	Leaf/ethanol	*Biomphalaria pfeifferi*	864.10	[97]
	Fruit/methanol	*Bulinus globosus*	1.33	
*Zanha goluogensis* Hiern	Stem/ethanol	*Biomphalaria glabrata*	60	[96]

**Table 5:** Egyptian species with activity against *S. mansoni* by Yousif et al. [95].

Plant Species	Part	LC_50_ (ppm)	LC_90_ (ppm)
Myrtaceae			
*Callistemon viminalis* (Soland. Ex Gaertn) Cheel	Leaves	6.56	9.49
	Branches	1.49	2.26
*C. rigidus* R. Br.	Aerial Roots	1.89	3.80
*C. speciosus* (Sims.) DC	Leaves/Branches	1.80	5.50
*C. citrinus* Stapf	Leaves	1.89	3.80
	Branches	1.80	4.10
*Eucalyptus citriodora* Hook	Bark	5.95	6.90
	Branches	10.00	11.07
*Eucalyptus rostrata* Dehnh.	Branches	7.80	13.50
*Eugenia edulis* Vell	Leaves	5.93	10.08
*E. javanica* Lam	Branches	9.70	12.90
*Melaleuca leucadendron* (L.) L.	Leaves	1.90	2.50
	Branches	2.30	6.20
*M. stypheloides* Sm.	Leaves/Branches	4.80	8.70
Asclepiadaceae			
*Cryptostegia grandiflora* R. Br.	Branches	11.40	23.80
Cruciferae			
*Zilla spinosa* (L.) Prantl	Fruits	10.50	48.90
Moraceae			
*Ficus trijuja* L.	Branches	14.40	39.50
Zygophylacae			
*Fagonia mollis* Delile	Herb	3.70	22.40

**Table 6:** Molluscicidal activities of medicinal plants from China against *Oncomelania hupensis*, the intermediate host of *Schistosomiasis japonica.*

Species Name	Plant Parts Used	Extraction Solvent	Activity (%Mortality/ LC or LD) Value	Active Compounds	References
*Acorus gramineus*	Rhizome, leaf	Ethanol	Exposure time = 72 h; Dose = 200 mg/L		[136]
			Rhizome: 75% mortality		
			Leaf: 56.25% mortality		
*Buddleja lindleyana*	Leaf	Ethanol	Ethanol: 100% mortality caused after 72 h exposure at a concentration of 200 mg/L	Acacetin-7-rutinoside:	[123,136]
		N-butanol for fractionations		LC_50_ = 36. 12 mg/L (24 h)	[124]
			N-butanol fractions:		
			Exposure time = 72 h; Dose = 50 mg/L	LC_50_ = 3.26 mg/L (72 h)	
			LC_50_ = 39.1 mg/L		
			LC_90_ = 59.28 mg/L		
*Clerodendron cyrtophyllum*	Branch, Leaf	Ethanol	Exposure time = 72 h; Dose = 200 mg/L		[136]
			Branch: 56.25% mortality		
			Leaf: 65% mortality		
*Eupatorium adenophorum*	leaf, roots and stems	Water	Leaf extract: 100% mortality caused after 82 h exposure with 0.27% (*w*/*v*) extract		[137]
			Roots extract: 56.7% mortality after 76 h with 0.86% (*w*/*v*) extract		
			Stem extract: 40 7% mortality after 82 h with 0.86% (*w*/*v*) extract		
*Ginkgo biloba*	sarcotesta granule	petroleum ether, ethyl acetate, ethanol	Petroleum ether: LC_50_ =7.81 mg/L	Ginkgolic acids isolated from pretroleum extract caused snail mortalities to be 45% (C13:0), 65% (C15:1), 0% (C17:1); exposure time = 72 h; concentration of extract = 2 mg/L	[138]
			Ethyl acetate: LC_50_ = 27.33 mg/L		
			Ethanol: LC_50_ = 64.14 mg/L		
	Leaf	Ethanol	Ethanol: 100% mortality caused after 72 h exposure at a concentration of 100 mg/L	Petroleum ether fractioned of the ethanolic extracts caused 100% mortality at a dose of 35 mg/L; exposure time: 72 h	[121]
*Hemerocallis fulva*	Root	Ethanol	76.25% mortality caused after 72 h at a concentration of 200 mg/L		[136]
*Herba agrimoniae*	Herb	Ethanol	80% mortality caused after 72 h exposure; dose = 100 mg/L		[121]
*Jatropha curcas*	Seeds	Water extract	Around 50% snail mortality caused from 48 exposure at a concentration of 0.03% (*w*/*v*)	phorbol esters	[139]
		Oil was produced from nuts by pressure and phorbol esters were enriched by extracting five time with an equal volume of methanol.			
*Juglis cathayensis* var. *formosana*	Fruit	Ethanol	60% mortality caused after 72 h exposure at a concentration of 100 mg/L		[136]
*Macleaya cordata* (Willd) R. Br	Leaf	Ethanol	1–7 alkaloid components were evaluated for molluscicidal activity.		[135]
		Total Alkaloid was extracted	Highest activity (73.33% mortality) observed after 48 h exposure with 28 mg/L of Alkaloid component 2		
			LC_50_ and LC_90_ values of Alkaloid component 2 = 6.35 and 121.23 mg/L, respectively		
*Nerium indicum* Mill	Leaf	N-butanol,	Exposure time: 48 h		[140]
		Water	N-butanol: LD_50_ =16.2 mg/L		
			Water: LD_50_ =13.2 mg/L		
*Peucedanum praerutorum*	Root	Ethanol	83.33% mortality caused after 72 h exposure; dose = 100 mg/L		[121]
*Pterocarya Stenoptera* DC	Leaf	N-butanol, Water	Exposure time = 48 h		[140]
			N-butanol: LD_50_ = 505.1 mg/L		
			Water: LD_50_ = 359.5 mg/L		
*Pulsatilla chinensis (Bunge) Regel*	Root	Water	LC_50_: 0.48 mg/L; exposure time = 24 h	hederacochiside C, hederacolchiside A1	[132,133,134]
*Rheum palmatum*	Root tubers	Water	> 50% snail mortality was achieved after 48 h exposure with 0.2% (*v*/*v*)	Anthraquinones including rhein, chrysophanol-anthron, rheum-emodin and physcion	[139]
*Rhumex dentatus*	Root tubers	Water	> 50% snail mortality was achieved after 48 h exposure with 0.2% (*v*/*v*)	Anthraquinones including rhein, chrysophanol-anthron, rheum-emodin and physcion	[139]
*Rhinacanthus nasutus*	herb	Ethanol	81.25% mortality caused after 72 h exposure with 200 mg/L extract		[136]
*Rumex japonicum*	Roots	N-butanol and Water crude extracts	Exposure time = 48 h		[140]
			N-butanol: LD_50_ =398.1 mg/L		
			Water: LD_50_ = 90.0 mg/L		
*Sapium sebiferum*	fruit	Ethanol	55% mortality caused after 72 h exposure with 200 mg/L extract		[136]
*Solanum xanthocarpum* (Schrad and Wendl)	Fruit	Ethanol	100% snail mortality was achieved after 48 h exposure with 4.321 mg/L extract		[111]
			LC_50_ = 0.181 mg/L for 72h of exposure		[141]
*Torreya grandis*	Leaf	Ethanol	80% mortality caused after 72 h exposure; dose= 100 mg/L		[121]
